# *Histoplasma* Virulence and Host Responses

**DOI:** 10.1155/2012/268123

**Published:** 2011-10-05

**Authors:** Mircea Radu Mihu, Joshua Daniel Nosanchuk

**Affiliations:** ^1^Department of Medicine, Sound Shore Medical Center of Westchester, New Rochelle, NY 10802, USA; ^2^Division of Infectious Diseases, Departments of Medicine and Microbiology and Immunology, Albert Einstein College of Medicine, Bronx, NY 10461, USA

## Abstract

*Histoplasma capsulatum* is the most prevalent cause of fungal respiratory disease. The disease extent and outcomes are the result of the complex interaction between the pathogen and a host's immune system. The focus of our paper consists in presenting the current knowledge regarding the multiple facets of the dynamic host-pathogen relationship in the context of the virulence arsenal displayed by the fungus and the innate and adaptive immune responses of the host.

## 1. Introduction

Histoplasmosis was first described in 1906 by Darling among the workers of the Panama Canal [1], and it is currently the most common cause of fungal respiratory disease with almost 500,000 individuals acquiring the fungus each year [2]. The etiologic agent responsible for histoplasmosis is *Histoplasma capsulatum*, a thermally dimorphic fungus with worldwide distribution. The fungus is primarily found in soil, where it exists in a mycelia form. In the United States, highly endemic areas include regions along the Mississippi and Ohio River valleys, where seroprevalence studies have shown that up to 80% of individuals are skin test positive for histoplasmin [3].

The entry portal of *H. capsulatum *is through inhalation of aerosolized of 2–4 *μ*m diameter microconidia [4]. Morphogenesis is initiated after infection with the conidia developing into a 2–4 *μ*m oval yeast form. The fungus is rapidly ingested by macrophages and neutrophils, but manages to avoid intracellular destruction. Intracellular yeast can be transported diffusely via the lymphatics and into the bloodstream. Nevertheless, initial infection is typically contained by innate and adaptive host responses. In immunocompetent individuals, the pulmonary disease is usually subclinical to limited, typically with flu-like symptoms, including fever, cough, headaches, and myalgias. However, lethal disease can occur in otherwise healthy individuals who acquire a large inoculum infection. Additionally, severe primary infection is more common and reactivation of latent infection occurs in immunocompromised persons, particularly in HIV-infected population and transplant recipients. Disseminated disease occurs in a small fraction of infected individuals, but this form of histoplasmosis continues to carry a high fatality rate even in patients receiving appropriate medical treatment [5]. More recently, treatments with inhibitors of tumor necrosis factor-*α* have been shown to place patients at high risk for developing histoplasmosis [6].

## 2. *H. capsulatum* Virulence Factors

The characterized virulence determinants of *H. capsulatum *are mainly surface expressed molecules that mediate the interaction between the fungus and the host's immune cells allowing the pathogen to evade destruction by innate immune response and facilitate the replication of the yeast in its new environment.

Heat shock protein 60 (HSP60), which has important roles in chaperoning intracellular proteins and supervising adequate protein folding, has also recently been described as an essential surface molecule, mediating the recognition and phagocytosis of the yeast by macrophages [7]. It acts as a ligand for CD11/CD18 macrophage receptor and, despite low number of antigenic sites, the coupling with the CR3 receptor is followed by rapid ingestion of the yeast. The interaction between *Histoplasma* and macrophage through HSP60 binding to CR3 results only in a mild host immune reaction, as it does not lead to a significant activation of phagocytes in the absence of other costimulatory signals [8]. This process allows the microorganism to survive and replicate inside the host cells [9, 10]. 

Heat shock protein 82 (HSP82) is another important molecule in normal development of *H. capsulatum* that also participates in the response to cellular stresses; it binds to a variety of cellular proteins, keeping them inactive until they have reached their proper intracellular location or have received the proper activation signal [11]. The role of HSP82 is further complicated by the thermal dimorphism displayed by *Histoplasma*, since changes in temperature represent both a stress inducer and a signal for yeast phase transformation. Recently, Edwards et al. demonstrated that a reduction in HSP82 decreases *Histoplasma* virulence in macrophages and severely impairs the fungus' ability to infect lungs in a murine infection model [12]. This suggests that a low basal level of HSP82 expression is sufficient only to preserve cellular functions at mammalian body temperature, but not to withstand other stresses encountered during infection. The temperature values during febrile episodes in the host, for example, represent one stress that requires HSP82 function, and defective mutant strains show a decreased ability to recover from transient in vitro incubation at 40°C. However, even at 37°C these defective mutants showed decreased virulence within macrophages, despite having identical growth in culture at similar temperature; this implies that HSP82 extends its role to enduring additional, nonthermal stresses during host infection. One example is the ability to survive oxidative stress, as shown by peroxide challenge studies [12]. 

YPS3 is a yeast phase-specific gene encountered in a subset of *H. capsulatum* strains. The encoded protein is found both as a fungal cell wall constituent and as a secreted molecule [13]. The exact function of this gene remains to be determined, but its importance in virulence is a certainty, since YPS3 mutants are attenuated in vivo [14].

The production of cell wall melanin is associated with virulence for diverse fungi; *H. capsulatum* conidia and yeast produce melanin or melanin-like pigments in vitro and yeast cells are melanized during mammalian infections [15]. The melanization process decreases the susceptibility of the fungus to amphotericin B and caspofungin and melanin can abrogate the potency of certain host defense mechanisms, such as free radicals and microbicidal peptides [16, 17].

Calcium-binding protein (CBP) represents another important factor in *Histoplasma* pathogenicity. CBP is secreted by the fungal cells during the yeast-phase of intracellular growth within the macrophage [18], and its importance for the virulence of the fungus was demonstrated both in vitro and in vivo. For example, CBP1 gene deletion yeast cells were rapidly cleared from the lungs of infected mice. Additionally, *H. capsulatum* growth is inhibited in limiting calcium conditions [19]. One of the hypotheses is that calcium acquisition represents an important factor for intracellular survival of the microorganism; another hypothesis targets the modulating effect of CBP in binding calcium to facilitate optimal phagolysosomal conditions for yeast growth.

Many *H. capsulatum *strains express *α*-(1,3)-glucan on their yeast cell surface. This polysaccharide forms a layer that conceals cell surface *β* glucans, which have antigenic properties, eluding the identification by the host phagocytic cells. The *β* glucan found in the cell wall of *Histoplasma* and other fungi is recognized by the Dectin-1 receptor on macrophages resulting in the triggered formation of reactive oxygen species and secretion of proinflammatory cytokines [20]. Confirmatory evidence for the role of the *α*-glucan was obtained using *Ags*-*1*-deficient *Histoplasma* mutant strains, where yeasts lacking the cell wall *α*-(1,3)-glucan were attenuated for virulence [21].

Histone 2B (H2B) has also been found to play a role in pathogenesis [22]. Histones are mainly intracellular components, but a study investigating passive immunity through administration of monoclonal antibodies from mice immunized with *Histoplasma *revealed antibody recognition of H2B present on the cell wall. The means by which historically intracellular-based molecules, as HSP60 or H2B, reach the yeast cell wall where they can interact with host cells was unclear until recently when macromolecular transport to the extracellular space was demonstrated to occur via vesicular secretion and active vesicular transport [23, 24]. 

Hydroxamate siderophores production by *Histoplasma *is another newly characterized virulence factor. Strains defective for the gene coding for siderophore production display impaired intracellular growth in both human and murine macrophages, which can be reversed by either exogenous iron addition or restoration of SID1 expression [25, 26].

## 3. Host Defense Mechanisms

After being exposed to* Histoplasma*, the host relies on both innate and adaptive immune response mechanisms to neutralize the pathogen and withstand infection. Macrophages and dendritic cells have major roles in the activation of cellular pathways, and the numerous cytokines, especially IFN-*γ* and TNF-*α*, significantly impact host responses.

Macrophages have a central role in the interaction between the fungus and the host, although their contribution has a dual nature. They represent the first line of defense during infection with *H. capsulatum*, as they rapidly phagocytose the inhaled conidia and transforming yeast cells, and the infected macrophage subsequently activate effector T cells and enhance the release of Th1-associated proinflammatory cytokines (IL-12, IFN-*γ*, and TNF-*α*) [27, 28]. Deprivation of zinc and iron is amongst the means used by macrophages to neutralize the intruding pathogen [29], along with production of superoxide, nitric oxide, and lysosomal hydrolysis. However, the fungus displays various mechanisms to elude destruction after phagocytosis. For example, *H. capsulatum *yeast cells are able to regulate the pH of the phagolysosomes at a neutral pH (approximately pH 6.5), where lysosomal hydrolases have decreased activity. Hence, the *H. capsulatum *yeast cells manage to survive and even replicate inside macrophages [30]. 

Dendritic cells are also an important effector of the innate immunity. They are able to phagocytose and degrade the fungal cells with higher efficacy than macrophages, which might be due to recognition of the pathogen via a different type of receptor (fibronectin receptor on dendritic cells versus CD18 on macrophages) [31]. Dendritic cells also are extremely efficient at processing and presenting antigens to specific CD8 T cells, either following ingestion of the yeast, or through “cross presentation” of fungal antigens engulfed from infected apoptotic macrophages [32]. In a recent study, the addition of antigen-presenting dendritic cells was found to suppress excessive production of IL-4 by CD4 T cells in lungs of CCR2-deficient mice infected with *H. capsulatum*, demonstrating the importance of these cells in the regulation of immune responses [33].

Cellular immunity is crucial in the host defense against intracellular pathogens; therefore, T cells, as the central effectors of the cellular immunity, have a substantial role in neutralizing *H. capsulatum *yeast cells. Mice depleted of both CD4 and CD8 T cells have accelerated time to death after challenge with *H. capsulatum *yeast cells, especially in a primary histoplasmosis model, which underlines the importance of the interaction between the two cell subsets in withstanding *Histoplasma* infection by eliciting a Th1 response [34]. CD4 cell depletion is associated with survival during primary infection, as a result of impaired IFN-*γ* production. The elimination of CD8 T cells results in decreased clearance of yeast cells in primary but not secondary infection. One particular subpopulation of T cells, V*β*4^+^ T cells, is preferentially expended during infection with *H. capsulatum,* and elimination of these cells from mice impairs their ability to resolve infection [35]. Th17 T cells and their interaction with regulatory T cells have recently been linked via the chemoattractant mediator CCR5 to the host's ability to effectively combat *H. capsulatum *infection; increases in Th17 cytokines and reductions in the number of regulatory T cells were associated with accelerated fungal clearance in CCR5-deficient animals [36]. 

Although cytokine responses are complex in histoplasmosis and alter over the course of disease, the main cytokines involved in *Histoplasma* clearance from the host are IL-12, IFN-*γ*, and TNF-*α* [34]. IL-12 through its ability to regulate IFN-*γ* production is critical in inducing a protective immune response in primary infection with the pathogen. IFN-*γ* is pivotal for the host's innate resistance to systemic infection with *H. capsulatum. *Survival of mice is significantly reduced in IFN-*γ*-deficient mice as well as in wild-type mice treated with neutralizing antibody to IFN-*γ* [37]. Patients with impaired IFN-*γ* signaling due to genetic defects are at increased risk for severe disease forms and administration of the cytokine can be therapeutic. For example, a report of recurrent disseminated *H. capsulatum* osteomyelitis in a patient with genetic IFN-*γ* receptor 1 deficiency describes progressive clearing of all bone lesions and normalization of inflammatory markers following subcutaneous therapy with IFN-*γ* [38]. Although IFN-*γ* is critical in primary infection, survival in secondary infection can be achieved in the absence of IFN-*γ*, as immunization of IFN-*γ*-deficient mice with an initial sublethal inoculum can prolong the survival of these mice when subsequently challenged with a high concentration of *H. capsulatum *yeast cells [39]. The major mechanism by which these mice were able to control secondary infection was through increased production of TNF-*α*. 

TNF-*α* is a key modulator of disease in both primary and secondary histoplasmosis, though different protective mechanisms are involved in these conditions [34, 40]. In primary infection, decreased survival of TNF-*α*-deficient mice has been attributed to an impaired ability to generate reactive nitrogen intermediates in the alveolar macrophages, although inducible nitric oxide synthase expression in lung tissue is preserved. During secondary infection, the increased mortality is largely due to a biased host reaction to a Th2-type response that is associated with elevated levels of both IL-4 and IL-10. These findings parallel the clinical data that clearly demonstrates that therapy with TNF-*α* inhibitors poses a significant increased risk for reactivation of latent histoplasmosis with a greater likelihood of severe, disseminated disease [8].

Humoral immune responses generally have a limited role in the clearance of intracellular pathogens; however, the protective role of antibodies against surface molecules of *H. capsulatum *has been described. Administration of monoclonal antibodies to *Histoplasma* H2B reduces fungal burden, decreases pulmonary inflammation, and prolongs survival in murine infection models [22]. The protective response was associated with increased levels of IL-4, IL-6, and IFN-*γ*. Similarly, antibodies to *H. capsulatum* HSP60 prolong the survival of the lethally infected animals [41, 42].

## 4. Discussion

Histoplasmosis is the most common endemic dimorphic fungal pathogen of man. The continuously expanding population of immunocompromised patients, secondary to the ongoing HIV epidemic, the increasing use of immunosuppressant therapies and rising number of transplant recipients, represents a high risk cohort for histoplasmosis. The mortality rate associated with invasive histoplasmosis is still unacceptably high, despite the use of broad spectrum antifungal agents, which emphasizes the need for developing novel therapies and effective preventive strategies.

As outlined in this paper, targeting virulence determinants of *H. capsulatum* and attempts to modify the capacity of the host to respond to the fungal invader are actively being pursued by researchers. Recent studies investigating the capacity of *H. capsulatum *to release a large number of proteins and other immunologically active compounds [23, 24, 43, 44] demonstrate the breadth of the fungus' ability to modify host responses. The high frequency of disease in the endemic areas and the increasing prevalence of the disseminated disease forms justify the development of adequate immunization strategies. Most recently, data has shown that vaccine-induced fungus-specific Th17 cells can confer protection against pulmonary histoplasmosis by recruiting and activating neutrophils and macrophages to the alveolar space [45]. Harnessing the host's existing armamentarium or supplementing the host's capacity, such as with the administration of cytokines or antibody to *H. capsulatum*, will be rich areas of study for the future.
